# Nutraceutical blends predict enhanced health via microbiota reshaping improving cytokines and life quality: a Brazilian double-blind randomized trial

**DOI:** 10.1038/s41598-024-61909-3

**Published:** 2024-05-15

**Authors:** Aline Boveto Santamarina, Jéssica Alves de Freitas, Lucas Augusto Moyses Franco, Victor Nehmi-Filho, Joyce Vanessa Fonseca, Roberta Cristina Martins, José Antônio Turri, Bruna Fernanda Rio Branco da Silva, Beatriz Emi Itikawa Fugi, Sumaia Sobral da Fonseca, Arianne Fagotti Gusmão, Eloísa Helena Ribeiro Olivieri, Erica de Souza, Silvia Costa, Ester Cerdeira Sabino, José Pinhata Otoch, Ana Flávia Marçal Pessoa

**Affiliations:** 1 Laboratório de Produtos e Derivados Naturais, Laboratório de Investigação Médica-26 (LIM-26), Departamento de Cirurgia, Faculdade de Medicina da Universidade de São Paulo, São Paulo, SP 01246903 Brazil; 2 Pesquisa e Desenvolvimento Efeom Nutrição S/A, São Paulo, SP 03317000 Brazil; 3 Laboratório de Parasitologia Médica (LIM-46), Departamento de Doenças Infecciosas e Parasitárias, Universidade de São Paulo Instituto de Medicina Tropical de São Paulo, São Paulo, SP 05403-000 Brazil; 4 Laboratório de Investigação Médica em Protozoologia, Bacteriologia e Resistência Antimicrobiana (LIM-49)Departamento de Doenças Infecciosas e Parasitárias, Universidade de São Paulo Instituto de Medicina Tropical de São Paulo, São Paulo, SP 05403-000 Brazil; 5 Grupo de Pesquisa em Economia da Saúde, Departamento de Ginecologia e Obstetrícia, Universidade de São Paulo Faculdade de Medicina, São Paulo, SP 01246903 Brazil; 6 Laboratório Interdisciplinar em Fisiologia e Exercício, Universidade Federal de São Paulo (UNIFESP), Santos, SP 11015-020 Brazil; 7 Graduação em Nutrição, Faculdade de Saúde Pública, Universidade de São Paulo, São Paulo, SP 01246904 Brazil; 8 International Research Center, A.C. Camargo Cancer Center, São Paulo, SP 01508-010 Brazil; 9 Ambulatório Monte Azul, São Paulo, SP 05801-110 Brazil; 10 Faculdade de Medicina da, Universidade de São Paulo, Hospital Universitário da Universidade de São Paulo, São Paulo, SP 05508-000 Brazil

**Keywords:** Phase I trials, Immunology, Cytokines, Microbiology, Endocrine system and metabolic diseases, Predictive markers, Health care, Nutrition, Quality of life, Weight management

## Abstract

Nutraceutical interventions supporting microbiota and eliciting clinical improvements in metabolic diseases have grown significantly. Chronic stress, gut dysbiosis, and metainflammation have emerged as key factors intertwined with sleep disorders, consequently exacerbating the decline in quality of life. This study aimed to assess the effects of two nutraceutical formulations containing prebiotics (fructooligosaccharides (FOS), galactooligosaccharides (GOS), yeast β-glucans), minerals (Mg, Se, Zn), and the herbal medicine *Silybum marianum* L. Gaertn., Asteraceae (Milk thistle or Silymarin). These formulations, namely NSupple (without silymarin) and NSupple_*Silybum* (with silymarin) were tested over 180 days in overweight/obese volunteers from Brazil's southeastern region. We accessed fecal gut microbiota by partial 16S rRNA sequences; cytokines expression by CBA; anthropometrics, quality of life and sleep, as well as metabolic and hormonal parameters, at baseline (T0) and 180 days (T180) post-supplementation. Results demonstrated gut microbiota reshaping at phyla, genera, and species level post-supplementation. The Bacteroidetes phylum, *Bacteroides*, and *Prevotella* genera were positively modulated especially in the NSupple_*Silybum* group. Gut microbiota modulation was associated with improved sleep patterns, quality-of-life perception, cytokines expression, and anthropometric parameters post-supplementation. Our findings suggest that the nutraceutical blends positively enhance cardiometabolic and inflammatory markers. Particularly, NSupple_*Silybum* modulated microbiota composition, underscoring its potential significance in ameliorating metabolic dysregulation. Clinical trial registry number: NCT04810572. 23/03/2021.

## Introduction

Gut health^1^, a balanced diet^2,3^, and a good quality of life are meaningful and interlaced^1^. Several factors play a crucial role in physical and mental well-being, contributing to a good quality of life. Adequate sleep^4^, a healthy diet^5^, and regular physical activity^6^ are essential components, besides genetic^7^, and environmental factors^8^. These elements collectively contribute to fostering a high quality of life.

Managing these factors often requires substantial lifestyle changes. However, keeping a healthy lifestyle is increasingly challenging nowadays, especially regarding diet and good sleep quality^9^. External factors, such as disruptions in the body's internal clock^10^, over-stimulating environments^11,12^, dietary habits^13,14^,and gut dysbiosis^15–17^, might directly affect sleep quality. Furthermore, intrinsic factors such as the neuro-immune-endocrine axis, which encompasses neuroendocrine elements like the hypothalamic–pituitary–adrenal (HPA) axis, and the enteric nervous system (ENS), along with immune factors typified by the sympathoadrenal axis (gut-lymphoid connection)^18^; play a crucial role in regulating sleep quality, immune response, and hormonal balance^15,19^. These factors interact with the intestinal microbiota, further influencing various physiological functions.^20,21^.

While asleep, critical immune system repair and regeneration processes occur^20,21^. Imbalances in the immune system, such as chronic inflammation, unbalanced IL-6 and TNF-α levels, and cortisol levels^15,19,22^, can lead to sleep disorders^20^. Properly managing stress hormones like cortisol is also a key factor in promoting good sleep patterns, since cortisol levels increase during the night, falling after 9 a.m. Thus, the inflammatory and hormonal disturbances caused by chronic stress can harm sleep quality and lead to sleep disorders^23^. High levels of IL-6 and cortisol contribute to disrupting the homeostasis of the circadian cycle, as their secretions are intricately linked to circadian cycle regulation^22^. Chronic stress is known as a risk factor for developing metabolic and mood disorders like anxiety and depression^24^. Research findings indicate that the gut microbiota is crucial in the onset and progression of inflammatory and psychiatric disorders, likely through gut microbiota-brain interactions and neuroinflammation^25^. The gut microbiota interacts with the central nervous system (CNS) via the gut-brain axis, impacting sleep regulation^15^. Microbial byproducts with anti-inflammatory potential like short-chain fatty acids (SCFAs) as well as microbiota-derived neurotransmitters like serotonin, acetylcholine, histamine, norepinephrine, dopamine, and gamma-aminobutyric acid^26^, act on the microbiota-gut-brain axis and are involved in the sleep pathways; thus imbalances in gut microbiota composition may trigger sleep disturbances or disorders^27,28^.

Regarding the gut microbiota composition, it is crucial to highlight the reduction in Bacteroidetes phylum abundance associated with sleep disorders^17^ and the enhancement of sleep quality^28,29^. Especially the *Bacteroides* and *Prevotella* genera abundance has been associated with an improvement in metabolites renowned as good sleep quality promoters^16,17,29,30^. Also, the *Alistipes onderdonkii* species, included in the Bacteroidetes phylum, has recently gained attention as a microbiota marker associated with inflammation and mental health implications, besides its role in cardiometabolic protection^30,31^.

Acknowledging these complexities, efforts to find promoters of long-term quality of life have emerged. These non-pharmacological strategies aim to enhance sleep and dietary quality by favoring gut microbiota balance. Notably, certain nutrients^32,33^ and natural compounds seem to play a key role in promoting the microbiota-brain-endocrine axis. Silymarin shows antioxidant and anti-inflammatory effects closely linked to the increase of neurotransmitters like serotonin and dopamine, which are involved in improving depression symptoms^34,35^ that could contribute to better sleep quality^34–36^. Additionally, zinc and magnesium work as crucial cofactors in the conversion of tryptophan amino acids to serotonin, which is closely associated with welfare, directly affecting the quality of life perception and sleep quality^37–39^. Given modern dietary deficiencies in fiber and essential minerals crucial for balanced gut microbiota, continuous intake of prebiotic fibers such as β-glucans, GOS (galactooligosaccharides), and FOS (fructooligosaccharides) may represent a nutritional strategy to enhance mineral absorption, maintain intestinal health, and fortify gut microbiota^40–42^.

Therefore, this randomized clinical trial aims to explore the effects of a 180-day consumption of nutraceutical compositions containing three sources of prebiotics (β-glucans, GOS, and FOS), minerals (zinc, magnesium, and selenium), and herbal medicine (Silymarin extract—*Silybum marianum* L. Gaertn) This nutritional and nutraceutical supplement has been created as a tool for the microbiota-brain-immune-endocrine axis modulation, with its primary objective being the enhancement of sleep quality and an overall improvement in quality of life through the reshaping of gut microbiota in overweight volunteers from southeastern Brazil.

## Results

### Anthropometric and biochemistry effects

Before the beginning of supplementation, the volunteers were screened for age, gender, body mass, height, and BMI which did not differ between groups stating the sample homogeneity. Regarding the anthropometric data, only the NSupple reduced the neck circumference, and only the *NSupple_Silybum* reduced the waist-to-hip ratio (WHR) and waist-to-height ratio (WHtR) after supplementation. Both supplements displayed reduced waist circumference (WC-mid). The diet intake parameters did not differ (Table 1).

**Table 1:** Demographic and anthropometric characterization, food intake, and serum parameters in the study population before and after the supplementation.

Group	NSupple	NSupple_*Silybum*					
Sample size (M/F)	21 (7/14)	20 (6/14)					
Age (years)	56 ± 6	57 ± 5					
Anthropometrics							

	NSupple			NSupple_*Silybum*			
Height (m)	1.622 ± 0.02			1.634 ± 0.02			
	T0	T180	*p*	T0	T180	*p*	
	Mean ± SD	Mean ± SD		Mean ± SD	Mean ± SD		
Body weight (kg)	72.24 ± 2.45	72.05 ± 2.38	–	74.07 ± 3.05	74.6 ± 3.11	–	
BMI (kg/m^2^)	27.45 ± 0.74	27.38 ± 0.73	–	27.55 ± 0.65	27.76 ± 0.73	–	
Neck (cm)	36.12 ± 0.63	35.54 ± 0.62	*0.0174*	36.13 ± 0.71	36.22 ± 0.82	–	
WC-mid (cm)	91.13 ± 1.70	89.87 ± 1.83	*0.0453*	92 ± 2.48	89.33 ± 2.47	*0.0268*	
WHR	0.87 ± 0.01	0.86 ± 0.01	–	0.88 ± 0.17	0.85 ± 0.16	*0.0062*	
WHtR	0.56 ± 0.01	0.55 ± 0.01	–	0.56 ± 0.012	0.55 ± 0.013	*0.0210*	
Dietary intake							
Energy (kJ)	5931 ± 1741	5679 ± 1403	–	6345 ± 1288	6942 ± 1994	–	
Carbohydrates (g)	62.48 ± 5.90	59.66 ± 9.22	–	61.48 ± 5.99	64.37 ± 9.94	–	
Fiber (g)	12.77 ± 6.603	12.15 ± 5.357	–	12.83 ± 6.196	11.39 ± 5.188	–	
Lipids (g)	13.86 ± 3.62	15.22 ± 3.32	–	15.80 ± 3.23	14.74 ± 4.75	–	
Proteins (g)	20.34 ± 5.10	21.91 ± 8.58	–	18.46 ± 1.85	17.94 ± 6.57	–	

Serum parameters							
	Reference values	T0	T180		T0	T180	
		Mean ± SD	Mean ± SD	*p*	Mean ± SD	Mean ± SD	*p*
Glycemia (mg/dL)	70–100 mg/dL	90 ± 1.93	95.95 ± 2.91	*0.0141*	88.85 ± 2.38	91.28 ± 2.54	–
HbA1c (%)	< 5.7%	5.29 ± 0.06	5.37 ± 0.06	*0.0484*	5.32 ± 0.12	5.411 ± 0.11	*0.0140*
IgA (mg/dL)	50–400 g/L	203.3 ± 18.31	179.1 ± 16.96	*0.0009*	237 ± 25.24	235.3 ± 25.28	–
C-RP (mg/dL)	0.3–1.0 mg/dL	0.1924 ± 0.02	0.1233 ± 0.02	*0.0201*	0.1725 ± 0.02	0.1369 ± 0.02	–
ALT (U/L)	4–36 U/L	9.095 ± 1.02	13.76 ± 1.17	*0.0023*	8.6 ± 3.43	11.22 ± 13.24	*0.0164*
AST/ALT ratio	< 1.67	2.337 ± 0.23	1.468 ± 0.07	*0.0014*	2.513 ± 0.22	1.897 ± 0.18	*0.0191*
TSH (mUI/L)	0.45–4.5 mUI/L	3.075 ± 0.37	2.212 ± 0.27	*0.0009*	2.857 ± 0.37	2.284 ± 0.27	–
Cortisol (ug/dL)	6.0–18.4 ug/dL	14.46 ± 0.90	11.66 ± 0.95	*0.016*	14.21 ± 0.86	12.42 ± 1.27	–
Cortisol/ C-RP ratio	0.3–1.0 mg/dL	0.819 ± 0.08	1.192 ± 0.21	*0.0369*	1.292 ± 0.25	1.312 ± 0.23	–

BMI (body mass index): < 18,5 underweight, 18.5–24.9 eutrophic, 25.0–29.9 overweight, > 30 obesity; Neck circumference: < 37 cm for men and < 34 cm for women; WC-mid (waist circumference in middle abdomen): < 102 cm for men and < 88 cm for women; WHR (waist-to-hip ratio): < 1.0 for men and < 0.85 for women; WHtR (waist-to-height ratio): < 0.5; C-RP: C-Reactive protein AST: aspartate aminotransferase; ALT: alanine aminotransferase; TSH: thyroid-stimulating hormone. Data values are expressed as mean ± SD.

The serum parameters evaluated show that only NSupple reduced IgA, TSH (thyroid stimulating hormone), C-reactive protein (C-RP), cortisol levels, and increased fasting glycemia and cortisol/C-RP ratio. The glycated hemoglobin (HbA1c) and ALT (alanine aminotransferase) levels were increased in both groups. Moreover, both groups reduced AST/ALT ratio after supplementation as shown in Table 1.

### Cytokines and chemokines modulation

The plasma cytokines and chemokines were accessed before and after supplementation, as shown in Table 2. The CCL5/RANTES, CXCL9/MIG, interferon-gamma (IFN-γ) levels, and IL-6/IL-10 ratio were reduced by both supplements after 180 days. Furthermore, after supplementation, both groups increased their interleukin 4 (IL-4) levels. The CXCL10/IP10 and IL12p70 levels increased solely in the NSupple group. The (tumor necrosis factor-alpha) TNF-α was reduced exclusively by the NSupple_*Silybum* supplement. The IL-6 level was increased in the NSupple group, while reduced in NSupple_*Silybum*. The CXCL8/IL-8, IL-1β, IL-10 levels, and TNF-α/IL-10 ratio did not differ.

**Table 2:** Cytokines and chemokines expression in overweight volunteers after 180 days of supplementation.

Group	NSupple					NSupple_*Silybum*				
	T0		T180		*p*	T0		T180		*p*
(pg/mL)	Mean ± SD	Min–Max	Mean ± SD	Min–Max		Mean ± SD	Min–Max	Mean ± SD	Min–Max	
IL-1β	2.48 ± 0.72	1.01–5.69	1.88 ± 0.53	0.01–4.28	*-*	2.42 ± 1.05	1.40–3.65	2.24 ± 0.63	1.26–2.67	*-*
IL-4	4.72 ± 1.02	0.01–14.01	6.06 ± 1.26	0.01–18.42	*0.017*	5.71 ± 1.44	0.01 -14.72	7.47 ± 2.37	1.16–26.79	*0.032*
IL-6	1.17 ± 0.13	1.01–1.47	1.36 ± 0.31	1.01–2.14	*0.022*	1.39 ± 0.18	1.14–1.55	1.14 ± 0.12	1.01–1.32	*0.016*
IL-10	2.72 ± 0.16	1.33–4.19	2.81 ± 0.20	2.04–5.16	*-*	2.54 ± 0.95	1.52–5.12	2.35 ± 0.38	1.61–2.91	*-*
IL-12p70	6.80 ± 0.52	2.93–13.51	8.72 ± 0.58	3.39–13.58	*0.002*	8.63 ± 3.19	4.98–17.32	8.87 ± 2.45	4.44–14.60	*-*
TNF-α	1.85 ± 0.28	1.32–2.42	1.85 ± 0.22	1.56–2.30	*-*	2.49 ± 1.12	1.80–5.92	2.10 ± 0.99	1.27–5.15	*0.001*
IFN-γ	3.44 ± 1.32	1.11–8.54	1.53 ± 0.72	0.01–4.62	*0.031*	7.88 ± 3.27	1.31–27.58	4.63 ± 2.31	0.01–14.95	*0.027*
CXCL10/IP-10	4.68 ± 0.68	1.85–12.30	7.09 ± 1.61	2.28–30.85	*0.030*	4.02 ± 1.66	1.95–6.73	4.18 ± 0.96	2.71 ± 5.74	*-*
CCL5/RANTES	42.62 ± 6.33	8.58–97.52	29.84 ± 4.51	0.01–69.29	*0.050*	36.5 ± 6.92	10.02–79.19	23.36 ± 2.39	12.43–36.04	*0.029*
CXCL9/MIG	133.2 ± 27.03	20.20–307.4	87.75 ± 15.25	26.79–197.1	*0.037*	136.5 ± 87.52	10.34–296.3	91.22 ± 60.66	18.79–206.3	*0.047*
CXCL8/IL-8	67.74 ± 8.85	18.14 -166.3	70.89 ± 7.55	27.54–156.7	*-*	65.28 ± 26.92	21.16–122.7	71.37 ± 24.66	24.48–106.5	*-*
IL-6/IL-10 ratio	0.45 ± 0.15	0.22–0.87	0.34 ± 0.21	0.01–0.60	*0.028*	0.58 ± 0.15	0.40–0.80	0.45 ± 0.12	0.27–0.61	*0.006*
TNF-α/IL-10 ratio	0.68 ± 0.05	0.51–0.97	0.82 ± 0.10	0.11–1.35	-	0.93 ± 0.38	0.46–1.75	0.77 ± 0.18	0.43–1.15	-

Values are expressed as pg/mL (mean ± standard deviation) in NSupple (n = 6–21) and NSupple_*Silybum* (n = 6–20).

### Sleep, mood, and quality of life effects

Volunteers were screened for chronotype before the supplementation establishing the majority as Intermediate chronotype. Concerning sleep quality, both supplements reduced the Epworth Sleepiness Scale (ESS) score. However, only the NSupple group reduced the Mini-Sleep Questionnaire (MSQ-BR) score after supplementation. Evaluating the Pittsburgh Sleep Quality Index (PSQI), the NSupple improved sleep quality (C1) with greater sleep latency (C2) scores. Also, exclusively the NSupple_*Silybum* reduced the Sleep medication use (C6) shown in Table 3. The competencies of PSQI, namely (C3) Sleep Duration, (C4) Sleep Efficiency, (C5) Sleep Disturbance, and (C7) Daytime Dysfunction as well as the PSQI Global score did not change post-supplementation (Table 3).

**Table 3:** Chronotype**,** Sleep quality, daytime sleepiness, quality of life, and mood characterization in the study population before and after the supplementation.

Group	NSupple	NSupple_*Silybum*								
Chronotype										
Definitely morning	14% (n = 3)	10% (n = 2)								
Moderately morning	24% (n = 5)	35% (n = 7)								
Intermediate	43% (n = 9)	40% (n = 8)								
Moderately evening	19% (n = 4)	15% (n = 3)								
Definitely evening	–	–								

Group	NSupple					NSupple_*Silybum*				
	T0		T180		*p*	T0		T180		*p*
	Mean ± SD	CI 95%	Mean ± SD	CI 95%		Mean ± SD	CI 95%	Mean ± SD	CI 95%	
Epworth sleepiness scale (ESS)										
ESS Total Score	10.23 ± 5.85	8.07–2.37	8.29 ± 5.02	6.44–10.13	*0.004*	10.45 ± 5.57	8.41–12.49	8.58 ± 6.14	6.33–10.83	*0.0104*
Mini-Sleep Questionnaire (MSQ-BR)										
MSQ-BR Score	31.31 ± 9.22	27.8–4.82	27.41 ± 8.61	24.14–30.69	*0.0051*	27.38 ± 7.46	24.69–30.06	25.91 ± 7.42	23.23–28.58	*–*
Pittsburgh Sleep Quality Index (PSQI)										
PSQI Global score	6.05 ± 2.90	5.00–7.13	5.16 ± 2.81	4.13–6.19	*–*	6.90 ± 3.79	5.51–8.29	6.35 ± 4.35	4.76–7.95	*–*
(C1) Sleep Quality	1.19 ± 0.87	0.87–1.51	0.77 ± 0.67	0.53–1.02	*0.0355*	1.19 ± 0.95	0.85–1.54	1.00 ± 0.93	0.65–1.34	*–*
(C2) Sleep Latency	0.71 ± 0.69	0.45–0.96	1.13 ± 1.02	0.75–1.50	*0.0412*	1.48 ± 1.12	1.07–1.89	1.13 ± 1.11	0.72–1.54	*–*
(C3) Sleep Duration	1.13 ± 1.02	0.75–1.50	1.13 ± 0.67	0.88–1.37	*–*	1.39 ± 1.02	1.01–1.76	1.23 ± 1.02	0.85–1.60	*–*
(C4) Sleep Efficiency	0.42 ± 0.81	0.12–0.71	0.42 ± 0.72	0.15–0.68	*–*	0.48 ± 0.96	0.13–0.84	0.45 ± 0.85	0.14–0.76	*–*
(C5) Sleep Disturbance	1.13 ± 0.56	0.92–1.33	1.03 ± 0.75	0.75–1.31	*–*	1.13 ± 0.11	0.90–1.36	0.94 ± 0.57	0.73–1.15	*–*
(C6) Sleep Medication	0.35 ± 0.95	0.00–0.70	0.26 ± 0.81	-0.04–0.56	*–*	0.65 ± 1.11	0.24–1.05	0.23 ± 0.76	-0.05–0.5	*0.0518*
(C7) Daytime Dysfunction	0.76 ± 0.58	0.53–0.97	0.62 ± 0.86	0.29–0.95	*-*	1.00 ± 0.97	0.645–1.35	1.39 ± 1.48	0.84–1.93	*-*
WHO Quality of Life-BREF										
Overall QoL and General health	3.50 ± 0.76	3.22–3.78	3.88 ± 0.49	3.70–4.07	*0.0093*	3.52 ± 0.61	3.29–3.74	3.88 ± 0.57	3.68–4.10	*0.0007*
Physical domain	3.78 ± 0.65	3.54–4.02	3.93 ± 0.51	3.75–4.12	*0.0508*	3.76 ± 0.57	3.55–3.97	3.87 ± 0.59	3.65–4.09	*-*
Psychological domain	3.70 ± 0.49	3.52–3.88	3.78 ± 0.57	3.57–3.98	-	3.54 ± 0.46	3.37–3.71	3.75 ± 0.46	3.59–3.92	*0.0053*
Social relationships	3.67 ± 0.58	3.48–3.85	3.74 ± 0.44	3.58–3.90	-	3.6 ± 0.70	3.43–3.94	3.76 ± 0.79	3.47–4.05	-
Environment	3.50 ± 0.51	3.31–3.69	3.56 ± 0.41	3.41–3.71	-	3.57 ± 0.47	3.40–3.74	3.64 ± 0.42	3.49–3.80	-
Brunel Mood Scale (BRUMS)										
BRUMS total score	12.74 ± 2.14	9.75–15.8	12.25 ± 1.81	9.8 -.14.7	-	12.31 ± 2.49	8.7–15.9	11.63 ± 2.23	8.6–14.6	-
Tension	4.35 ± 1.62	3.76–4.95	4.13 ± 1.73	3.49–4.76	-	4.19 ± 2.04	3.44–4.94	3.93 ± 1.80	3.27–4.59	
Depression	3.03 ± 2.66	2.05–4.01	2.84 ± 2.25	2.01–3.66	-	2.87 ± 3.07	1.74–3.99	2.61 ± 2.94	1.53–3.69	-
Anger	2.64 ± 2.33	1.79–3.50	2.16 ± 2.08	1.39–2.92	-	2.96 ± 3.25	1.77–4.16	2.58 ± 2.74	1.57–3.58	-
Confusion	4.25 ± 2.11	3.48–5.03	4.16 ± 1.55	3.59 -4.73	-	4.51 ± 2.41	3.63–5.40	4.13 ± 2.26	3.30–4.96	-
Vigor	4.93 ± 2.35	4.07–5.79	4.64 ± 2.10	3.87–5.41	-	5.74 ± 2.59	4.79–6.69	4.83 ± 2.66	3.86–5.81	*0.0271*
Fatigue	3.40 ± 1.75	2.74–4.05	3.60 ± 1.16	3.16–4.03	-	3.52 ± 1.59	2.91–4.12	3.21 ± 0.98	2.83–3.57	-

ESS > 10 represents daytime sleepiness at a potentially clinical level; MSQ-BR > 30 represents sleep disturbance; PSQI global score > 5 indicates poor sleep quality; WHO Overall QoL > 3 represents good quality of life perception; BRUMS total score > represents 24 mood disturbance.

Accessing the WHO Quality of Life score (WHOQoL-Bref) both supplements improved the Overall quality of life and general health perception after 180 days. Also, the NSupple group increased the physical domain perception while NSupple_*Silybum* improved the psychological domain perception after supplementation. The Social relationships and Environment domains in WHOQoL-Bref did not differ throughout time. Evaluating the Brunel Mood Scale (BRUMS) only the NSupple_*Silybum* impacted vigor perception after supplementation, the other mood variables did not differ (Table 3).

### Gut microbiota reshaping by the supplementation

**Figure 1:**
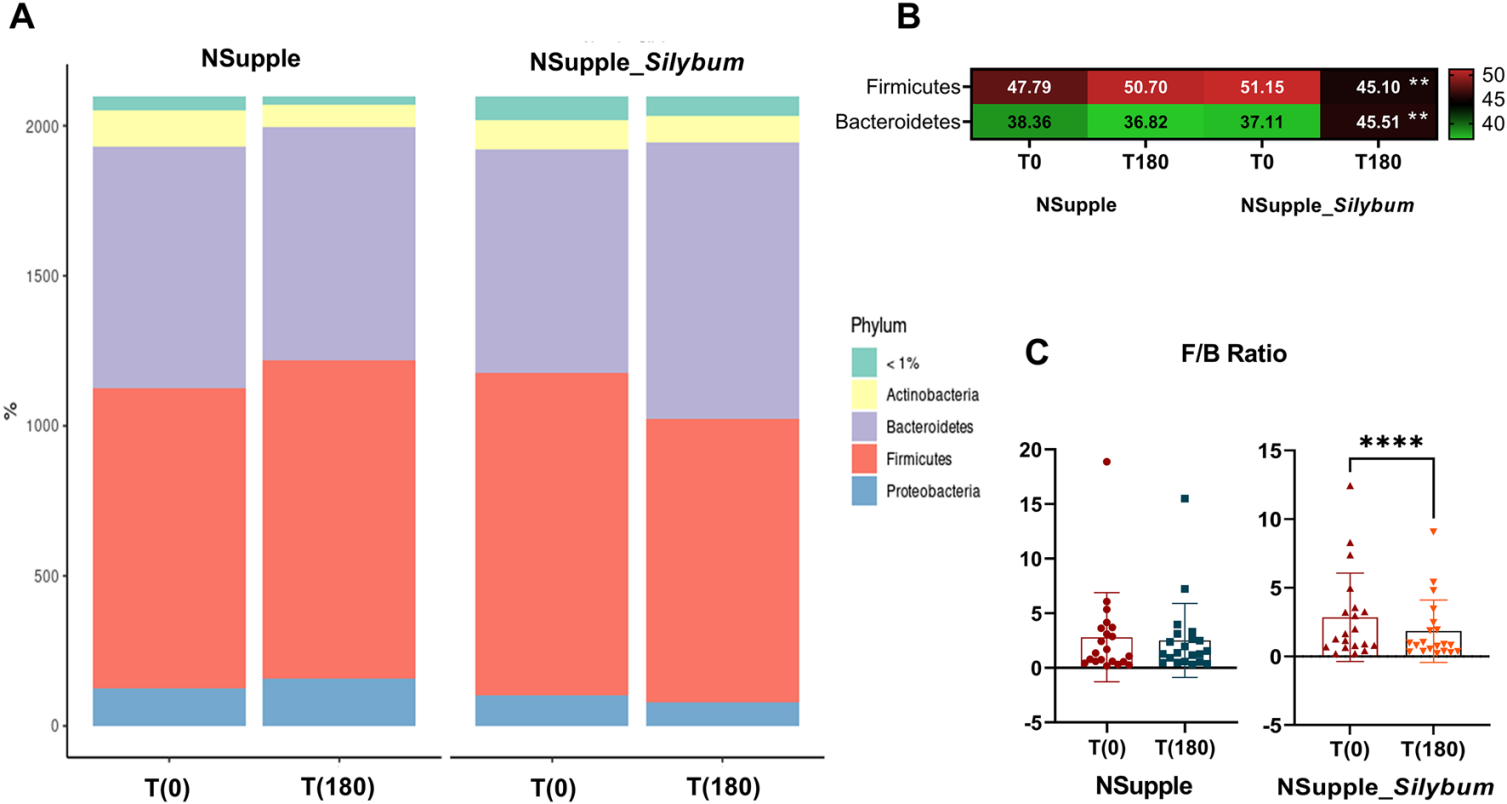
Phyla composition of the gut microbiota in overweight volunteers after 180 days of supplementation. Shown are the abundance of the phyla profile (**A**); heatmap of the microbial phyla abundance with statistical significance (**B**); and Firmicutes/Bacteroidetes ratio (F/B ratio) (**C**); from time zero [T(0)] and 180 days [T(180)] post-supplementation with NSupple (n = 21) and NSupple_*Silybum* (n = 20) in overweight volunteers. Values are expressed as the percent of relative abundance (mean ± standard deviation). *p < 0.05, **p < 0.01, ***p < 0.001, ***p < 0.001.

After 180 days, the gut microbiota phyla composition (Fig. 1A) in volunteers who underwent NSupple_*Silybum* supplementation demonstrated a relative decrease in Firmicutes and an increase in Bacteroidetes (Fig. 1B). Additionally, there was a noticeable reduction in the F/B ratio (Fig. 1C).

**Figure 2:**
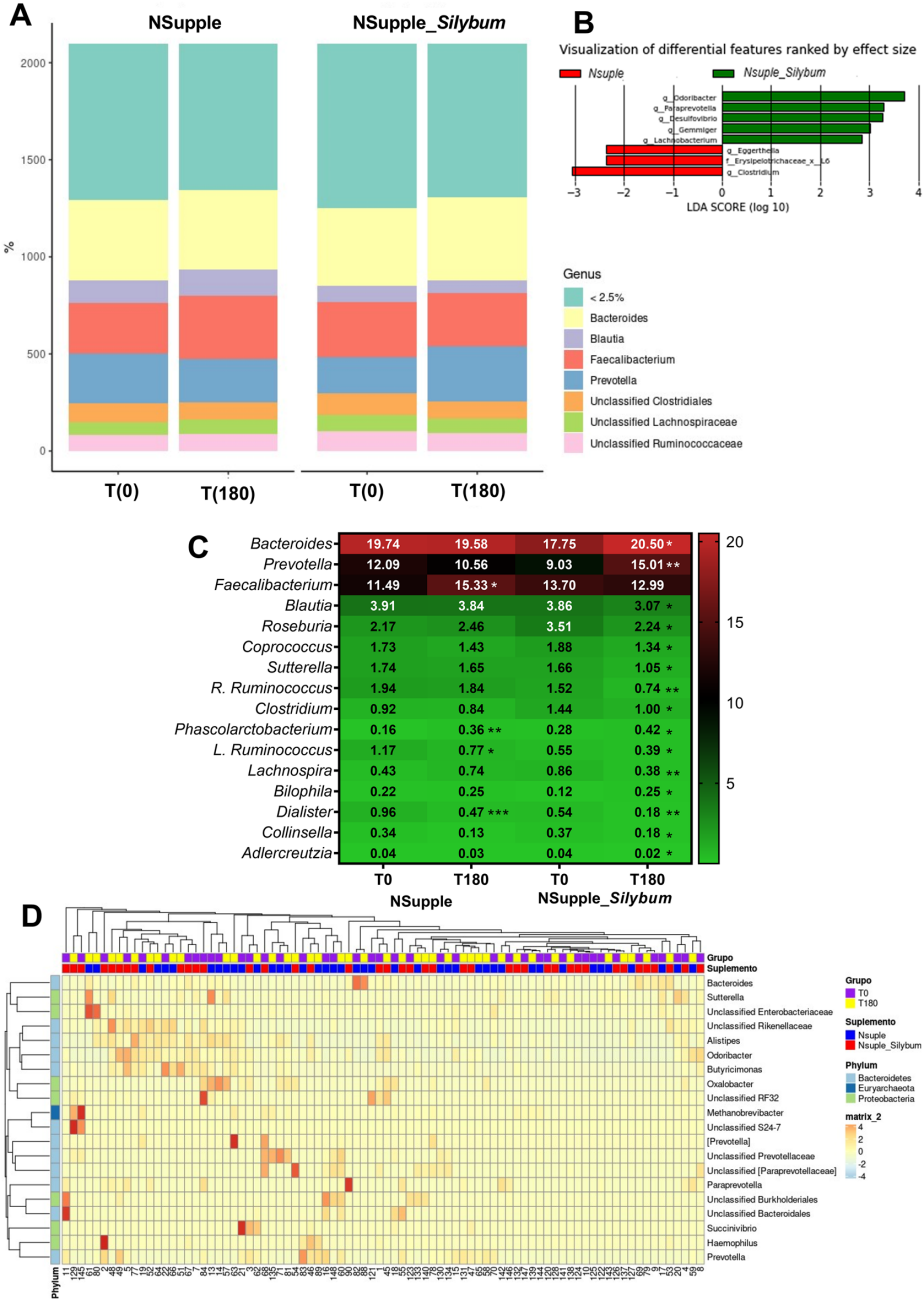
Genera composition of the gut microbiota in overweight volunteers after 180 days of supplementation. Shown is the abundance of the genera profile (**A**); The logarithmic linear discriminant analysis (LDA) effect size (LEfSe) scores. Red bars indicate that taxa were enriched in NSupple, and green bars indicate those that were enriched in NSupple_*Silybum*. Only the taxa with LDA > 2.0 are shown (**B**); heatmap of the microbial genera abundance with statistical significance (**C**); heatmap depicting taxonomic distribution readings of volunteers' microbiome. The figure highlights variations and groupings in the taxonomic characteristics of volunteers before (T0) in purple and after 180 days of supplementation (T180) in yellow, with NSupple, in blue, and NSupple_*Silybum*, in red. Each column represents the relative abundance, delineated by intensity profiles for individual samples. Colors on the map reveal the relative positioning of read count data; ranging from light blue to orange signifies values above the mean. The color tones denote the distance of each data point from the mean line. At the sidebar of the heatmap there is the overall relative abundance of the taxa at a given taxonomic level, represented by three phyla, Bacteroidetes, Euryarchaeota and Proteobacteria**,** for gut bacterial taxa from time zero [T(0)] and 180 days [T(180)] after supplementation (**D**); with NSupple (n = 21) and NSupple_*Silybum* (n = 20) in overweight volunteers. Values are expressed as the percent of relative abundance (mean ± standard deviation). *p < 0.05, **p < 0.01, ***p < 0.001.

The analysis of the genera profile in both groups has demonstrated a significant influence on the gut microbiota composition (Fig. 2A). In the LEfSe analysis, we identified that both supplements modulated eight distinct bacterial features, each achieving a Linear Discriminant Analysis (LDA) score higher than two (Fig. 2B). The NSupple_*Silybum* group appeared to markedly increase the abundance of statistically significant genera such as *Bacteroides* and *Prevotella*, while decreasing *R. Ruminococcus, Lachnospira,* and *Dialister* (Fig. 2C). To facilitate the comprehension of microbiome data, we utilized a heatmap visualization technique, specifically emphasizing the phylum and genera that displayed differential representation between the supplemented groups (Fig. 2D).

**Figure 3:**
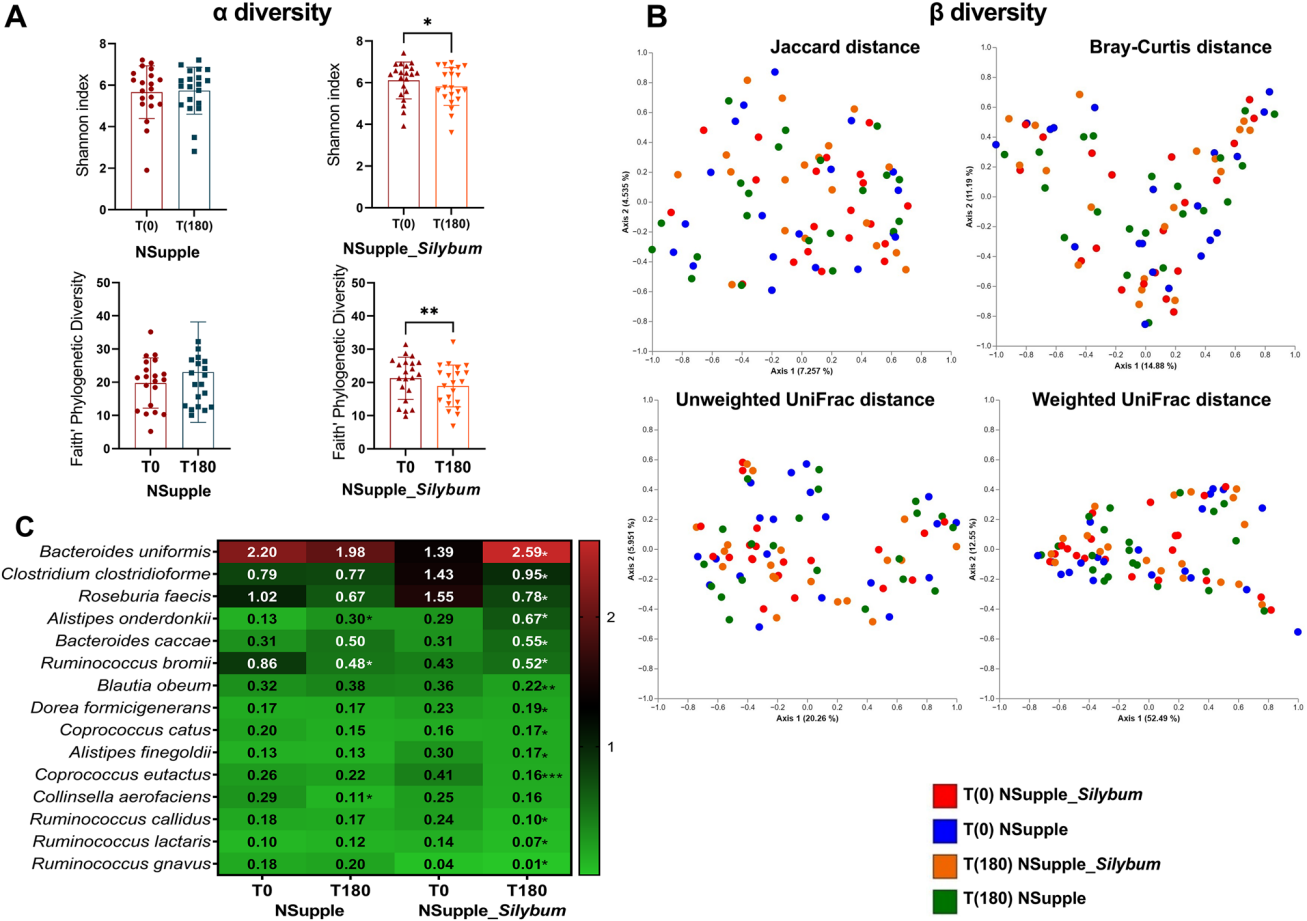
Diversity indices and Genera composition of the gut microbiota in overweight volunteers after 180 days of supplementation. Shown are the Alpha (α) diversity indices for species richness, (Shannon), and Phylogenetic diversity (Faith’s PD) (**A**), beta (β)- diversity Jaccard distance, Bray–Curtis distance, Unweighted, and Weighted UniFrac distances (**B**), and the heatmap of the microbial species abundance with statistical significance (**C**) from gut microbiota from time zero [T(0)] and 180 days [T(180)] after supplementation with NSupple (n = 21) and NSupple_*Silybum* (n = 20) supplement in overweight volunteers. Values are expressed as the percent of relative abundance (mean ± standard deviation). *p < 0.05, **p < 0.01, ***p < 0.001.

Among the alpha (α)-diversity indices, Shannon and Faith’s PD displayed a statistical reduction in microbial abundance within the NSupple_*Silybum* group post-supplementation (Fig. 3A). Additional (α)-diversity indices were computed; however, no statistically significant differences were observed. These results are provided in the supplementary material for further reference (Fig. S1). Similarly, there were no statistical differences in the realm of beta (β)-diversity assessment, which encompassed Jaccard, Bray–Curtis Unweighted UniFrac, and Weighted UniFrac distances. These findings were visually represented through the Principal Coordinate Analysis Plot (PCoA) (Fig. 3B).

In terms of the supplements' impact on the microbiota species abundance, the NSupple_*Silybum* group showed a discernible influence by increasing the abundance of specific gut microbiota species, including *Bacteroides uniformis, Alistipes onderdonkii*, and *Bacteroides caccae*. Concurrently, there was a decrease observed in *Roseburia faecis, Blautia obeum, Dorea formicigenerans*, and *Coprococcus eutactus* (Fig. 3C).

### Predictive parameters of microbiota modulation

The logistic regression data in Table 4 shows that improvement of WC-IC was associated with an increase in Bacteroidetes phyla and *Bacteroides* genus abundance in the NSupple_*Silybum* group. Also, ESS improvement was associated with the enrichment of *Faecalibacterium* in the NSupple_*Silybum* group.

**Table 4:** Regression analysis from gut microbiota association with clinical-demographic characteristics after supplementation.

Group		%	R^2^	IC95% min	IC95% max	*p*
Logistic regression						
NSupple_*Silybum*	WC-IC (cm)	Bacteroidetes	8.33	1.038	67.14	*0.046*
		*Bacteroides*	8.33	1.038	67.14	*0.046*
	ESS	*Faecalibacterium*	13.5	1.47	123.74	*0.021*
*Multiple linear regression*						
Group		%	Coef	IC95% min	IC95% max	*p*
NSupple	↓ AST/ALT ratio	Bacteroidetes	− 0.43	− 0.16	− 0.70	*0.003*
		*Sutterella*	3.00	4.55	1.44	*0.001*
	↓ ESS	*Collinsella*	0.58	0.89	0.27	*0.002*
		*Bacteroides*	− 0.21	− 0.04	− 0.37	*0.017*
		*L. Ruminococcus*	0.42	0.68	0.16	*0.010*
	↑ Overall QoL and General health score	*Faecalibacterium*	0.08	0.04	0.12	*0.001*
	↓ IL-6/IL-10 ratio	*Bacteroides caccae*	0.14	0.28	0.00	*0.047*
NSupple_*Silybum*	↓ WC-IC (cm)	*Bacteroides*	− 0.32	− 0.03	− 0.61	*0.031*
		*Dorea*	8.09	15.41	0.77	*0.032*
		*Roseburia*	1.29	2.15	0.43	*0.005*
		*Sutterella*	1.89	3.21	0.57	*0.008*
	↓ WHtR	*Bacteroides*	− 0.002	0.000	− 0.004	*0.028*
		*Dorea*	0.047	0.092	0.003	*0.039*
		*Roseburia*	0.008	0.013	0.003	*0.006*
		*Sutterella*	0.012	0.020	0.004	*0.006*
	↓ AST/ALT ratio	*Phascolarctobacterium*	− 19.49	− 1.15	− 37.83	*0.039*
		*Eubacterium biforme*	7.62	10.80	4.43	0.001
	↓ ESS	*Alistipes onderdonkii*	2.57	3.92	1.22	0.001
	↑ Overall QoL and General health score	*Bilophila*	0.57	0.07	1.07	0.028

The multiple linear regression analysis shows the association of a decrease in the AST/ALT ratio with reduced Bacteroidetes phyla (Coef 0.43) and a 3% increase in *Sutterella* genus abundance in the NSupple group. Still in the NSupple group, a decrease in ESS score was associated with reduced *Bacteroides* genus (Coef − 0.32) as well as increased *Collinsella* genus (Coef 0.58) and *L. Ruminococcus* species (Coef 0.42). The increased Overall QoL and General health perception were associated with the raised *Faecalibacterium* genus (Coef 0.08) in the NSupple group. Also, the reduction in IL-6/IL-10 ratio was associated with increased *Bacteroides caccae* (Coef 0.14) species in the gut microbiota of the NSupple group as shown in Table 4*.*

Regarding the NSupple_*Silybum* group, both anthropometric parameters reduction of WC-IC and WHtR were associated with the same microbiota genera modulation. For each centimeter of reduction in WC-IC there was a 0.32% reduction in the *Bacteroides* genus abundance; an 8.09% increase in the *Dorea* genus; a 1.29% increase in the *Roseburia* genus; and a 1.89% increase in the *Sutturella* genus. Similarly, the reduction in WHtR was associated with a reduction in the abundance of the genus *Bacteroides* (Coef − 0.002), and an increase in the genera *Dorea* (Coef 0.05), *Roseburia* (Coef 0.008) and *Sutturella* (Coef 0.012).

In the NSupple_*Silybum* group, the AST/ALT ratio reduction was also associated with a 19.49% decrease in the *Phascolarctobacterium* genus and a 7.62% increase in the abundance of *Eubacterium biforme*. Moreover, the improvement of ESS has been associated with a 2.57% increase in *Alistipes onderdonkii* species. Also, the rise in Overall QoL and General health perception was associated with a 0.57% increase in the *Bilophila* genus in the NSupple_*Silybum* group (Table 4).

## Discussion

The advancement of delving natural supplements focused on supporting long-term health is progressively expanding. Maintaining a well-balanced state of health might lower the long-term risk of illness and has encouraged the exploration of well-being promoter therapies^43^. Recently, research on gut microbiota modulation has gained prominence by applying prebiotics, probiotics, phenolic compounds, macronutrients, and micronutrients attempting to achieve the ideal microbiota for health^44^. Recently, the gut microbiota has emerged as a pivotal regulator of several physiological systems, including the neuro-immune-endocrine axis, among others. Given its wide impact on health, alterations in the microbiota, like dysbiosis, might trigger metabolic issues like obesity and type 2 diabetes, as well as, sleep disorders, and a significant disturbance in mood and quality of life^28^. This has led to an increasing chase for comprehensive investigations into the effects of new combinations of nutrients on gut health. Thus, this clinical trial investigates a specific blend of nutrients, comprising prebiotics (β-glucans, GOS, and FOS), essential minerals (zinc, magnesium, and selenium), and an herbal medicine extract (Silymarin from *Silybum marianum* L. Gaertn., Asteraceae). on metabolic, endocrine, and behavioral enhancements associated with gut microbiota adaptability.

The methodological approach aimed to characterize the gut microbiota among overweight and sedentary volunteers living in southeastern Brazil before and after their intake of two different nutraceutical supplements over 180 days. Hence, we assessed whether the microbiota alterations could correlate with the improvement of chronic inflammation, sleep disorders, and gut dysbiosis commonly observed in chronic stress and metabolic diseases. Although our volunteers were categorized as overweight rather than obese, we observed enhancements in anthropometric measurements in the NSupple_*Silybum* post-supplementation linked to sleep disturbances, and central adiposity. The literature shows a close relationship between the improvement of anthropometrics, metabolic markers, sleep duration^45^, and obstructive sleep apnea syndrome^46^. The results presented here are promising initially showing the potential of the nutraceutical blend as a modulator of endocrine, metabolic, and anthropometric parameters without diet or exercise interventions.

It's noteworthy that the decrease in anthropometric parameters aids as indicators of a decreased cardiometabolic risk as well as in sleep patterns change. The reduction in WC-mid in both groups, neck circumference in NSupple, WHR, and WHtR in NSupple_*Silybum* depict visceral adiposity, which is widely recognized as more beneficial for metabolic health than simply reducing overall body mass or BMI. These findings are even more interesting if we highlight the absence of lifestyle modifications during supplement consumption. It allows us to suggest a lower risk of metabolic syndrome, type 2 diabetes, cardiovascular diseases, and other metabolic disorders among overweight people under supplementation^47^. Liu et al. 2017 have shown that FOS and GOS supplementation impaired fasting glucose and glycemic response in OGTT, which might relate to increased HbA1c, using machine learning and LEfSe analysis. The authors state that FOS and GOS might favor the microbiota modulation hindering the butyrate-producer genera such as *Ruminococcus* and *Coprococcus*^48^ which agrees with our microbiota findings. Butyrate, a short-chain fatty acid produced by microbiota, might improve type 2 diabetes features by increasing glucagon-like peptide-1 (GLP-1), an incretin hormone that participates in glucose homeostasis recovering fasting glycemia, insulin resistance, and inflammation^49^. Thus, the literature allows us to suggest that the prebiotics in our supplementation reduced butyrate production by the microbiota reshape leading to an increase in fasting glycemia and HbA1c. Nevertheless, it is important to note that prebiotics such as FOS and GOS have a dubious impact on glucose metabolism requiring in-depth research^50,51^. Despite a rise in fasting glycemia in the NSupple group and HbA1c in both groups post-supplementation, the parameters remained under the reference values range, thus not rendering the use of the supplements unfeasible.

Confirming the positive metabolic outcomes, in this clinical trial we note the improvement of liver function by a decrease in the AST/ALT ratio in both supplementation groups. These findings agree with the metabolic enhancements observed in volunteers reducing the onset of metabolic disorders alongside body changes^52,53^, these results boost the applicability and potency of the supplement blend tested. Also, prior preclinical investigations on this nutrient composition showcased a similar hepatoprotective effect, characterized by diminished liver enzymes and AST/ALT ratio. The preclinical model also provided insight into this hepatoprotective action, attributing it to increased mitochondrial biogenesis activity in the liver reducing metabolic dysfunction-associated fatty liver disease (MAFLD) in the mouse model^54^. This evidence strongly suggests a parallel with our clinical trial, where the improvement in the AST/ALT ratio, and the decrease in visceral adiposity measurements could be linked to the mitochondrial activity promoted by the supplementation. Enhanced mitochondrial activity provides a compelling rationale for the reduction in these metabolic and anthropometric parameters without alterations in dietary habits or an increase in physical activity.

These endocrine and metabolic factors are linked to alterations in sleep patterns, potentially resulting in sleep disorders that directly influence individual perception of quality of life and mood throughout the day, which might hinder performance in daily activities^55^. Our results have shown improvement in sleep quality perception by the MSQ and enhanced sleep quality and latency in PSQI scores in the NSupple group post-supplementation. Also, both groups reduced day sleepiness as shown in ESS. These results might impact the improved perception of overall quality of life and general health in both groups especially related to physical in the NSupple group and psychological aspects in the NSupple_*Silybum* group. All these changes may be connected since cross-sectional studies indicate a noteworthy inverse relationship between sleep duration and waist circumference and WHR (waist-to-hip ratio), suggesting that shorter durations of sleep tend to coincide with central adiposity^45^.

Obesity and high visceral adiposity markers are recognized as risk factors for sleep disorders such as obstructive sleep apnea syndrome^56^ an important sleep disorder that affects the quality of life and promotes pro-inflammatory cytokines expression (interleukin 6/IL-6 and tumor necrosis factor-α/TNF-α) impacting metabolic diseases onset^46,57–59^. These conditions involve changes in serum alanine aminotransferase (ALT) and aspartate aminotransferase (AST), as well as nonalcoholic fatty liver disease (NAFLD). This association is tied to the chronic, intermittent periods of low oxygen levels during apnea episodes, leading to liver inflammation and fibrosis, closely linking sleep disorders and metabolic alterations^60^.

Sleep disorders are also associated with high cortisol levels^61^ which contribute to immune and inflammation dysregulation of pro-inflammatory factors such as IL-6, TNF- α, and C-reactive protein (C-RP). In sleep disorders such as insomnia, the increase in cortisol may be a marker of CRH (Corticotropin-releasing hormone) and norepinephrine activity during the night, resulting in non-restorative sleep and prolonged wakefulness^61^. Our results show the reduction in pro-inflammatory markers like C-RP and cortisol in the NSupple group demonstrating a positive effect on biological stress markers related to sleep quality^62^. Also, the Cortisol/C-RP ratio serves as a valuable marker for assessing the negative feedback loop between the hypothalamic–pituitary–adrenal (HPA) axis and the inflammatory response system in major mood disorders like depression and anxiety. Studies on mood disorders indicate that individuals exhibiting high depression symptoms have a reduced CORT/C-RP ratio—suggesting inadequate cortisol release relative to elevated C-RP and tend to experience worse stress-induced negative mood reactions^63,64^. Thus, even though our sample did not present mood disorders diagnostics, the increase in the Cortisol/C-RP ratio in the NSupple group might be suggested as a positive secondary outcome of the supplementation on mood disturbs reflecting on overall quality of life perception^65^.

During the study, the volunteers consistently maintained an approximate intake of 50% of the recommended daily fiber amount, ranging between 25 and 50 g^66^. Notably, this percentage remained unchanged even upon the inclusion of the supplements. Recognizing the pivotal role of sustaining the health and equilibrium of the intestinal microbiota, our focus has pivoted towards acknowledging nutrients and their derivatives as key elements for enhancing the gut microbiome.

Prebiotics fibers and flavonolignans in the *Silybum marianum* (L.) Gaertn. (silymarin) demonstrate direct efficacy in improving the gut physiology, microbiota diversity, and abundance in a metabolic syndrome mice model^15^. Prebiotic fibers are known for their benefits in providing energy and improving the microbiota diversity and abundance in mice^67^ and humans^68,69^, however always in high doses. Anon, the flavonolignans show a “phytotherapic prebiotic” effect by its chemical structure. Flavonoids, and lignans, provide carbohydrates, amino acids, and other compounds for microbiota fermentation and metabolism, like prebiotic fibers^70–73^. Furthermore, minerals such as zinc^74^, magnesium, and selenium^75^ serve as vital cofactors in fortifying the intestinal epithelium and immune system, thereby influencing various metabolic pathways conducive to enhancing intestinal health.

The supplement NSupple_*Silybum* emerged as the most impactful in modulating the abundance of the intestinal microbiota, notably promoting homeostasis by eliciting a reduction in Firmicutes and an increase in Bacteroidetes phyla. Moreover, the NSupple_*Silybum* effectively lowered the Firmicutes-to-Bacteroidetes (F/B) ratio. Lněničková and colleagues showed an increase in the *Bacteroides* genus after silymarin supplementation in humans, which supports our findings^72^.

Specifically, the influence of NSupple_*Silybum* on the Bacteroidetes phylum extended to the *Bacteroides* genus, particularly when correlated with decreased WC-IC (waist circumference—iliac crest) measurements and WHtR (waist-to-height ratio). These findings underscore the anti-inflammatory and cardioprotective attributes, and with the microbiota abundance inherent in the silymarin-containing supplement, concurrently demonstrating an improvement in sleep quality, as previously mentioned. Notably, the *Faecalibacterium* genus has exhibited a linked association with enhanced sleep quality^76^, aligning with our results in the NSupple_*Silybum* group. A groundbreaking discovery of our investigation lies in delineating a robust association between *Alistipes onderdonkii* species, belonging to the Bacteroidetes phylum, and improved sleep quality results in the NSupple_*Silybum* group. Despite its classification as a commensal organism within the large intestine^77^, *Alistipes onderdonkii* has often been implicated in various disease states or intestinal disorders^31^.

Both supplement groups exhibited notable systemic anti-inflammatory effects following a 180-day supplementation period, characterized by a reduction in chemokines closely linked to metabolic and intestinal disorders. Specifically, levels of RANTES^40^, CXCL9/MIG^78^, IL-6, and the IL-6/IL-10 ratio, recognized markers associated with inflammatory diseases such as gastric cancer^79^, witnessed a decrease in both groups. Concurrently, there was an elevation in IL-4 in both supplement groups, renowned for its anti-inflammatory properties^80^. This modulation indicates a propensity towards mitigating inflammatory responses.

Nevertheless, NSupple_*Silybum* showcased more pronounced anti-inflammatory attributes in this regard. It remarkably decreased the levels of TNF-α, which is linked to sleep disorders. Furthermore, NSupple revealed a robust association between the higher presence of *Bacteroides caccae,* a gut commensal species able to degrade host-derived mucin glycans producing short-chain fatty acids (SCFAs)^81^, known for the anti-inflammatory effect, intestinal barrier recovery^82^, safeguarding the intestinal barrier^83^, and the reduction in the IL-6/IL-10 ratio. These anti-inflammatory properties reveal the supplement's therapeutic efficacy and hint at their differential impacts on specific inflammatory pathways and associated physiological responses.

When scrutinizing the effects of supplements concerning genera-based disparities, discernible positive impacts emerged. Prevotella is typically linked to diets abundant in plant-derived fibers, non-western carbohydrate profiles^84^, and flavonoid fermentation^85^, which might be related to the NSupple_*Silybum* effect. This genus, known for its anti-obesogenic attributes through propionate production^86^, demonstrated marked benefits following the administration of NSupple_*Silybum*. Among the 16 genera manifesting statistically significant differences, the intervention with NSupple accounted for alterations in merely 4 genera. In contrast, NSupple_*Silybum* displayed a substantial influence, modulating 12 of these genera. This highlighted the significant impact of flavolignan within NSupple_*Silybum* on the overall microbial composition. Our research delved into the isolated effects of Silymarin, as elucidated in our previous preclinical study. However, when administered in isolation, Silymarin failed to replicate the effects witnessed when it was combined with other nutrients within the supplement composition^42^. This underscores the symbiotic interplay among various components, emphasizing their collective contribution to the achieved outcomes. It reiterates the significance of the holistic synergy among supplement constituents in yielding positive effects.

In the evaluation of bacterial species, it's essential to initially recognize their predominantly commensal nature, acting as holobionts, essential for environmental eubiosis. Dysbiosis often facilitates opportunistic or pathogenic species growth. However, it's crucial to acknowledge that this classification is reliant on predictive scenarios, heavily influenced by the specific strain's attributes to determining a species as a probiotic or opportunist. In our study, we observed significant modulation of two pivotal species by NSupple_*Silybum*. An increased abundance of *Bacteroides uniformis*, a species renowned for its metabolic and immunomodulatory functions^87^, and a reduced abundance of *Clostridium clostridioforme* were observed in NSupple_*Silybum* group. Although considered commensal, *C. clostridioforme* is acknowledged as a reservoir for antimicrobial resistance^88^.

Nevertheless, several limitations were notable within our study. Firstly, the absence of a placebo control group restricted our ability to effectively discern the specific effects of the supplements administered, isolating the placebo effect. Secondly, the study grappled with a limited sample size, compromising the statistical power and generalizability of the findings. Thirdly, an absence of comprehensive metabolomic analysis on the fecal samples hindered our capacity to elucidate the intricate pathways and mechanisms contributing to the observed effects. These limitations must be considered in future studies addressing other populations and, in-depth mechanisms of action investigations.

Despite these limitations, the results underscored the potential of the NSupple_*Silybum* supplement. Notably, it enhances sleep quality and mitigates inflammation, primarily through the modulation of bacteria within the Bacteroidetes phylum. These initial findings suggest the supplement like a promising tool for preventive and therapeutic strategies in chronic non-communicable diseases and sleep disturbance through gut microbiota modulation.

## Methods

### Ethics committee approval

This research adhered to the Declaration of Helsinki and was formally approved by the Ethics Committee for the Analysis of Research Projects (CAPPesq) from the HC-FMUSP Research Ethics Committee under CAAE number 39984320.5.0000.0068. This study has been approved by the Brazilian National System of Genetic Registration (SisGen) under registration number AC29D69. Additionally, it was registered under the identification number NCT04810572 at ClinicalTrials.gov on 23/03/2021.

### Enrollment of participants and experimental design

**Figure 4:**
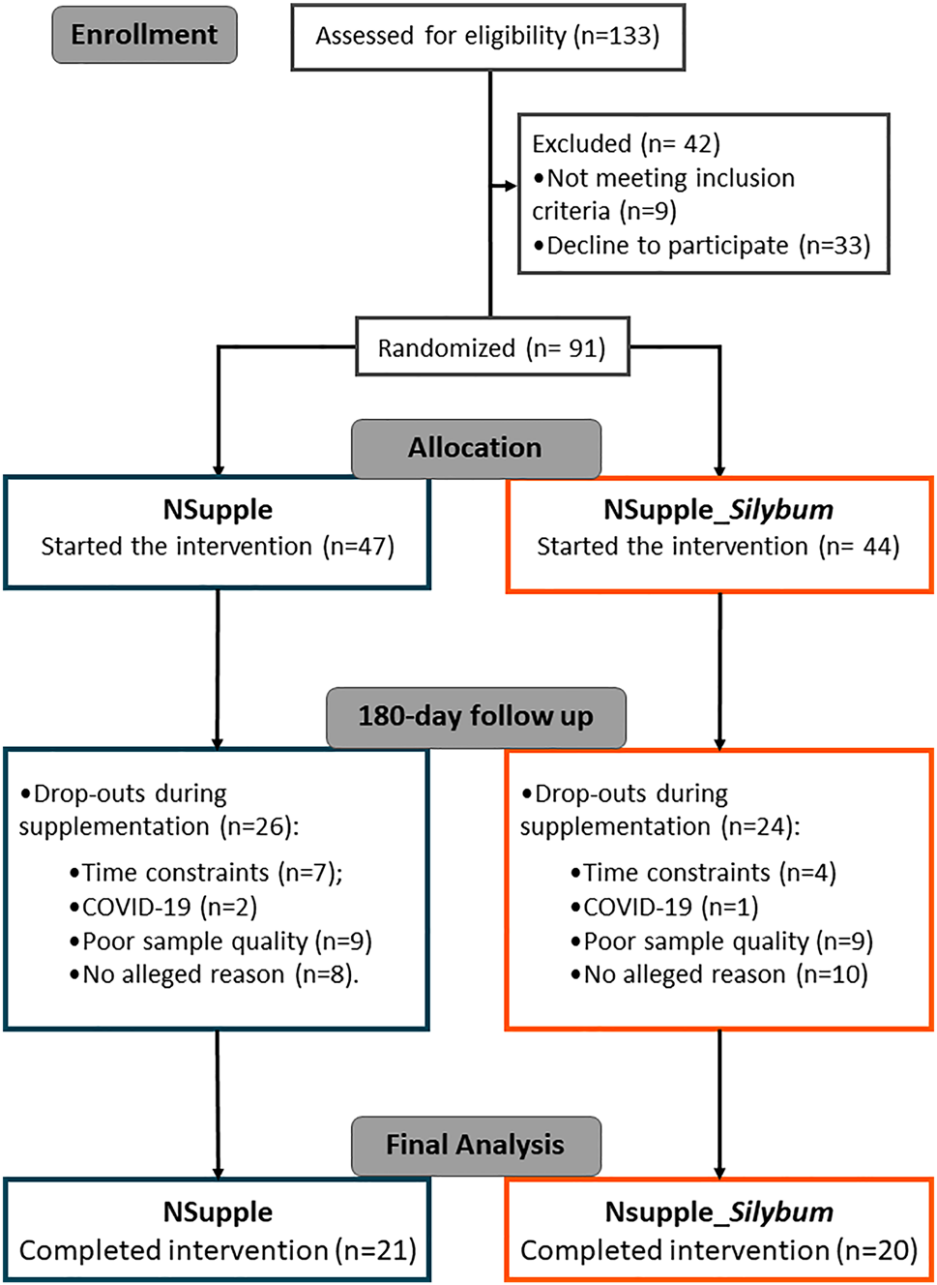
Consolidated Standards of Reporting Trials (CONSORT) flowchart describing the recruitment of volunteers and the experimental design carried out.

This clinical trial recruited 133 volunteer residents in the southeastern region of Brazil, at the outpatient clinic “*Ambulatório Monte Azul”* (São Paulo, Brazil), and through online advertisement from 03/01/2021 to 06/09/2021. The trial data collection ended on 12/20/2021. This double-blind, randomized trial evaluated participants at baseline (T0) and after 180 days of supplementation (T180). The trial adhered to pre-established protocols for inclusion/exclusion criteria, and supplementation previously described by Nehmi-Filho et al., (2023)^89^. Briefly, the exclusion criteria were: use of insulin injection, corticoids, and non-steroidal anti-inflammatory drugs for more than 15 days; AIDS or hepatitis diagnosis; pregnancy; and patients under chemotherapy treatment. For volunteers who met the research inclusion criteria, we applied stratified randomization considering factors such as age, sex, and BMI. The Consolidated Standards of Reporting Trials (CONSORT)^90^ flow chart outlines the trial's progression in Fig. 4. In summary, 133 individuals initially responded to the volunteer call. Subsequently, 42 individuals withdrew before randomization because they did not meet the inclusion criteria (n = 9) or declined to participate further (n = 33). Consequently, 91 individuals were randomized into two experimental groups: NSupple (n = 47) and NSupple_*Silybum* (n = 44) at T0. Throughout the 180-day supplementation period, 26 volunteers from the NSupple group were excluded due to time constraints (n = 7), COVID-19 infection (n = 2), poor sample quality (n = 9), or by no alleged reasons (n = 8). Similarly, 24 volunteers from the NSupple_*Silybum* group withdrew because of time constraints (n = 4), COVID-19 infection (n = 1), poor sample quality (n = 9), or unspecified reasons (n = 10). Consequently, by the end of the experiment, samples from 21 individuals in the NSupple group and 20 individuals in the NSupple_*Silybum* group which completed the 180 days of supplementation were analyzed. Blood and fecal samples were collected at both T0 and T180. All participants provided free and informed consent before participation.

### Nutraceutical composition formulas

Two different formulations were tested herein (Patent number BR 10 2020 016156 3) as previously published by Nehmi-Filho, Santamarina, et al*.,* 2023^89^ and described below. NSupple: zinc (Zn) 1%, magnesium (Mg) 1% (Purifarma Distribuidora Química e Farmacêutica, São Paulo, Brazil), Fructooligosaccharide (FOS) 45% (NutraFlora®, Westchester, Illinois, USA), selenomethionine (Se) 0.01%, Galactooligosaccharide (GOS) 10%, tixosil 5%, and 1.3/1.6-(β-glycosidic bonds) yeast β-glucans (*Saccharomyces cerevisiae*) 6% (Biorigin, São Paulo, Brazil); and NSupple_*Silybum:* zinc (Zn) 1%, magnesium (Mg) 1% (Purifarma Distribuidora Química e Farmacêutica, São Paulo, Brazil), Fructooligosaccharide (FOSs) 45% (NutraFlora®, Westchester, Illinois, USA), selenomethionine (Se) 0.01%, Galactooligosaccharide (GOS) 10%, tixosil 5%, 1.3/1.6-(β-glycosidic bonds) yeast β-glucans (*Saccharomyces cerevisiae*) 6% (Biorigin, São Paulo, Brazil), and *Silybum marianum* (3.11% of seed extract) (SM Empreendimento Farmacêutica LTDA, São Paulo, Brazil). The “*Solis Magistral Farmácia Homeopatia Sensitiva”* (São Paulo, Brazil) team was responsible for the study blinding, and preparing the formulations following EFSA guidelines^91^.

The absence of a placebo group might be unusual in the experimental design of a clinical trial, however, the literature discusses that the need for a placebo is pertinent only to drug trials^92^. Nevertheless, most clinical trials usually apply a placebo to facilitate outcome blinding and to control the placebo effect. However, especially research on gut microbiota must be careful with placebo application since most common placebos applied interact with the gut microbiota which might represent a bias. Starch, resistant starch, cellulose, sugar, and maltodextrin are common placebos generally considered inert for humans. However, they may be metabolized reshaping gut microbiota, producing metabolites used by host intestinal cells, and reaching the bloodstream, potentially offering beneficial effects^93–101^. Based on our literature review, there is no ideal placebo that did not interact with bacteria in microbiota studies^93–101^. Thus, we opted to avoid the placebo, using individual self-control instead, by comparing before and after supplementation in paired analyses.

### Volunteers’ anthropometrics

Anthropometric measurements were assessed at the initial time point (T0) and post-supplementation (T180). The volunteers' body mass and height were measured using the Body Composition Scale 2 (Xiaomi Mi, Beijing, China). Circumferences of the neck (cm), and waist (cm), were gauged using a plastic tape measure. Body Mass Index [BMI = body mass (kg)/height (m)^2^], waist-to-hip ratio (WHR), and waist-to-height ratio (WHtR) were calculated. The hip (cm) and iliac crest (cm) circumferences were measured and presented in supplementary data (Table S1).

### Serum parameter

Blood samples obtained at T0 and T180 between 7:00 a.m. and 9:00 a.m. underwent analysis for glycemia, HbA1c, IgA, albumin, C-reactive protein (C-RP), ALT, TSH, and cortisol. The AST/ALT ratio and Cortisol/C-RP were calculated. Additionally, Insulin, HOMA-IR, total cholesterol and fractions (HDL, LDL, VLDL, non-HDL), triglycerides, IgG, IgM, total protein, AST, alkaline phosphatase, creatinine, *gamma*-GT, and thyroxine were investigated and presented in supplementary data Table S1. These analyses were conducted by the *"Fleury Medicina e Saúde"* laboratory (São Paulo, Brazil).

### Dietary intake and physical activity data

Participants' dietary intake data were obtained at T0 and T180 from a three-day food diary and analyzed using DietPro software (version 6.1). Physical activity was assessed through the International Physical Activity Questionnaire (IPAQ), categorizing activities based on intensity^102^.

### Cytokines and chemokines levels

The cytometric bead array (CBA) test was employed to quantify cytokine and chemokines in plasma samples stored at − 80 °C. Beads, standards, reagents, and plasma samples were prepared according to the manufacturer's guidelines. Results were obtained using the BD Accuri flow cytometer and commercially available kits (BDTM Cytometric Bead Array) and commercial kits from BD™ Cytometric Bead Array (CBA) for “Human Chemokine” and “Human Inflammatory Cytokines” were used (BD Biosciences, USA). Standard dilution methods were applied for cytokine standards. Calibration of the flow cytometer with cytometer setup beads preceded the assay. Data analysis was performed in FCAP Array v2.0 software (SoftFlow, Pecs, Hungary), presenting results in pg/mL.

### Questionnaires for sleep, mood, and quality of life characterization

The Brazilian Portuguese Version of the Mini-Sleep Questionnaire (MSQ-BR) was used to assess the subjective quality of sleep^103^. Epworth sleepiness scale (ESS) was applied to measure the general daytime sleepiness level^104^. The Portuguese version of the Horne and Ostberg morningness-eveningness questionnaire was used to establish the volunteers' chronotype^105^.

The Pittsburgh Sleep Quality Index (PSQI)^103^ was applied to assess sleep quality and disturbances over 1 month. To assess the participants’ perception of quality of life, we applied the “World Health Organization quality of life instrument-short form” (WHOQoL-BREF)^106^. Brunel Mood Scale (BRUMS) was used as a validated instrument to screen the volunteer's mood perception^107^.

### Microbiome analysis

#### Sample collection and genomic extraction

Each participant collected approximately 1 g of feces and placed it in a guanidine stool preservation medium, to preserve the microbiome promptly after collection. The samples were maintained under temperature control, ranging from 2 to 8 °C during transport and 0 to − 80 °C during storage^108^. Genomic samples were obtained by extracting genomic material using about 0.25 g of feces and the DNeasy PowerSoil Kit (Qiagen, Germantown, MD, USA). The extracted material was then stored at − 20 °C until the library preparation phase.

#### Library preparation and sequencing

The library preparation and sequencing procedures are extensively detailed by Nehmi-Filho, et al.^42^. In summary, for prokaryotic community analysis, 16S rRNA (V4 region) sequences were directly amplified and sequenced using 515F/806R. This amplification was carried out using a bacterial/archaeal primer set, specifically 515F/806R^109^. The sequencing process was performed according to the manufacturer's instructions (Thermo Fisher Scientific, Waltham, Massachusetts, USA) using the Ion Chef System and the Ion S5 platform.

### Bioinformatic analysis

The detailed bioinformatic analysis was previously described by Nehmi-Filho, et al.^42^. Briefly, the 16S rRNA gene data underwent preprocessing and diversity estimation using Quantitative Insights Into Microbial Ecology (QIIME 2) version 2020.11^110^. The average number of sequences per sample was 52,389. The data were denoised with DADA2 (via q2‐dada2) using default parameters, which included a length threshold of 200 bp and an average quality Phred score of ≥ 30. This denoising step generated amplicon sequence variants (ASVs)^111^. The analysis identified 3428 ASVs. Following the construction of a phylogenetic tree, alpha and beta diversity metrics were calculated using Q2-diversity. The samples were rarefied to 19,803 sequences per sample^112^ before estimating these metrics.

The taxonomic classification of ASVs was performed using the Q2‐feature‐classifier^113^, specifically employing the naive Bayes classifier against the Greengenes 13_8 99% OTUs (Operational Taxonomic Unit) reference sequences^114^. The composition of microbiota communities was summarized at various taxonomy levels, including species, genera, families, orders, classes, and phyla ranks.

Additionally, alpha diversity metrics such as Chao1, Simpson, OTUs, Pielou's evenness, Shannon diversity, and Faith's phylogenetic diversity were calculated. The beta diversity metrics employed were Jaccard distance, Bray–Curtis distance, Unweighted and Weighted UniFrac distances. To enhance the interpretability of microbiome data, we employed a heatmap visualization technique focusing on phyla, genera, and species. Differential abundance analysis was conducted utilizing the R (4.3.1) package DESeq2 (1.42.0).

### Statistical analysis

The sample size was determined using G*Power 3.1 software^115^, assuming a T-test (Means: Difference between two dependent means-matched pairs) with (α) level of 0.05, a power (β) of 0.90, and an effect size (success rate) of 0.5. The calculated total sample size required was 36 volunteers. Considering potential dropouts, we increased the sample size by 20%, resulting in an ideal sample size of 43 volunteers to ensure data reliability. Data were classified as parametric or nonparametric based on the Shapiro–Wilks and Smirnov-Kolmogorov test and outliers were excluded by the Grubbs test. Continuous parametric data were shown as mean ± standard deviation, and nonparametric as median and interquartile range. Continuous parametric variables were performed Student T-Test and, for nonparametric variables, the Wilcoxon test was applied. Linear and logistic regression tests were also performed for continuous and categorical variables, respectively, to verify all variables associated with the outcomes of interest investigated in this study. For the linear regressions, the magnitudes of the differences between the baseline and final results were stipulated for each variable of interest in comparison with the dependent variable. For the logistic regressions, the normality thresholds were verified for each variable of interest when present and researched in the scientific literature, or when absent, the lower and upper thresholds were defined to the 50% quartile for all variables to be investigated. Analyses were performed using STATA® 16-SE (Stata Corp. LCC, College Station, TX, USA) and GraphPad Prism 9.0 (GraphPad Software, La Jolla, CA, USA) software. The genera that were differentially represented between supplement groups were determined using the R (4.3.1) package DESeq2 (1.42.0). Differential gene expression analysis based on the negative binomial distribution^116^. To determine the taxonomic characteristics most likely to explain differences between periods and supplement groups, we employed the algorithm Linear discriminant analysis Effect Size (LEfSe 1.1.2)^117^. For all analyses, significance was determined as p ≤ 0.05.

### Supplementary Information

Supplementary Information.

## Data Availability

The data that support the findings of this study are available from Ana Flávia Marçal Pessoa but restrictions apply to the availability of these data, which were used under license for the current study, and so are not publicly available. Data are however available from the authors upon reasonable request and with permission of Ana Flávia Marçal Pessoa. The datasets generated and/or analyzed during the current study are available in the GenBank ® repository, Bioproject PRJNA941000. Link: https://www.ncbi.nlm.nih.gov/sra/PRJNA941000 (Release date: 06/30/2025). The data that support the findings of this study are available on request from the corresponding author. This link is exclusive for the reviewers: https://dataview.ncbi.nlm.nih.gov/object/PRJNA941000?reviewer=lfevhd0p2jn9358imrei528946.
