# Supplementary material for: Nutraceutical blends predict enhanced health via microbiota reshaping improving cytokines and life quality: a Brazilian double-blind randomized trial
Source: Sci Rep. 2024 May 15;14:11127. doi: 10.1038/s41598-024-61909-3 (PMC11096337; doi:10.1038/s41598-024-61909-3)
Supplement: Supplementary file 1 — Supplementary Information. [file 41598_2024_61909_MOESM1_ESM.docx]

**Supplementary data**

**Table 1S.** Population physical activity level is classified by the International Physical Activity Questionnaire (IPAQ), anthropometrics, and serum profile characterization of the study population.

| Group | NSupple | | | NSupple_*Silybum* | | |
| --- | --- | --- | --- | --- | --- | --- |
|  | **T0** | **T180** |  | **T0** | **T180** |  |
| International Physical Activity Questionnaire (IPAQ) | | | | | | |
|  | n (%) | n (%) | *p* | n (%) | n (%) | *p* |
| Sedentary | 4 (19) | 4 (19) | *-* | 3 (15) | 4 (20) | *-* |
| irregularly active | 8 (38) | 7 (33) | *-* | 8 (40) | 7 (35) | *-* |
| Moderate active | 8 (38) | 8 (38) | *-* | 7 (35) | 7 (35) | *-* |
| High activity | 1 (5) | 2 (10) | *-* | 2 (10) | 2 (10) | *-* |
| Anthropometrics | | | | | | |
|  | Mean±SD | Mean±SD | *p* | Mean±SD | Mean±SD | *p* |
| Hip (cm) | 104.3±1.34 | 103.9±1.57 | *-* | 103.9±1.47 | 104.5±1.57 | *-* |
| WC-IC (cm) | 98.79±1.52 | 100.1±1.88 | *-* | 99.70±2.03 | 99.18±1.99 | *-* |
| Serum profile | | | | | | |
| Insulin (mU/L) | 11.81±1.25 | 11.67±1.16 | *-* | 12.15±1.49 | 12.11±1.52 | - |
| HOMA-IR | 2.6±0.26 | 2.767±0.29 | - | 2.75±0.41 | 2.589±0.34 | - |
| Total Cholesterol (mg/dL) | 225.1±6.67 | 219.6±7.55 | - | 191.2±9.12 | 191.2±9.79 | - |
| HDL-C (mg/dL) | 53±2.51 | 51.86±2.81 | - | 49.55±3.01 | 47.95±2.92 | - |
| LDL-C (mg/dL) | 142.2±5.24 | 140.9±6.54 | - | 117.8±8.28 | 119.1±8,83 | - |
| VLDL-C (mg/dL) | 24.72±0.87 | 26.86±1.99 | - | 23.8±1.40 | 22.58±1.79 | - |
| Non-HDL-C (mg/dL) | 172.1±6.66 | 167.8±7.44 | - | 141.6±8.54 | 143.2±8.64 | - |
| Triglycerides (mg/dL) | 127.1±6.15 | 134.9±11.43 | - | 123.5±9.23 | 137±13.24 | - |
| IgG (mg/dL) | 1134±48.76 | 1125±47.73 | - | 1071±41.58 | 1074±31.69 | - |
| IgM (mg/dL) | 112.9±10.75 | 101.9±1.18 | - | 118.4±10.73 | 111.2±9.41 | - |
| Total protein (g/dL) | 7.176±0.11 | 7.21±0.08 | - | 7.16±0.06 | 7.263±0.06 | - |
| AST (U/L) | 19.86±1.17 | 20.14±1.59 | - | 21.4±1.71 | 18.89±0.93 | - |
| Alkaline phosphatase (U/L) | 76.95±4.63 | 77.19±4.95 | - | 69.9±5.09 | 74.21±4.87 | - |
| Creatinine (mg/dL) | 0.8419±0.03 | 0.8224±0.03 | - | 0.844±0.03 | 0.8295±0.04 | - |
| *Gamma*-GT (U/L) | 19.72±2.57 | 19.72±2.37 | - | 25±3.43 | 23.47±2.78 | - |
| Thyroxine (ng/dL) | 0.9±0.03 | 0.9857±0.07 | - | 0.9588±0.02 | 0.9316±0.04 | - |
| Albumin (g/dL) | 4.519±0.06 | 4.605±0.03 |  | 4.58±0.04 | 4.65±0.05 |  |

WC-IC: waist circumference in iliac crest; AST: aspartate aminotransferase; IgG: Imunoglobulina G; IgM: Imunoglobulina G.


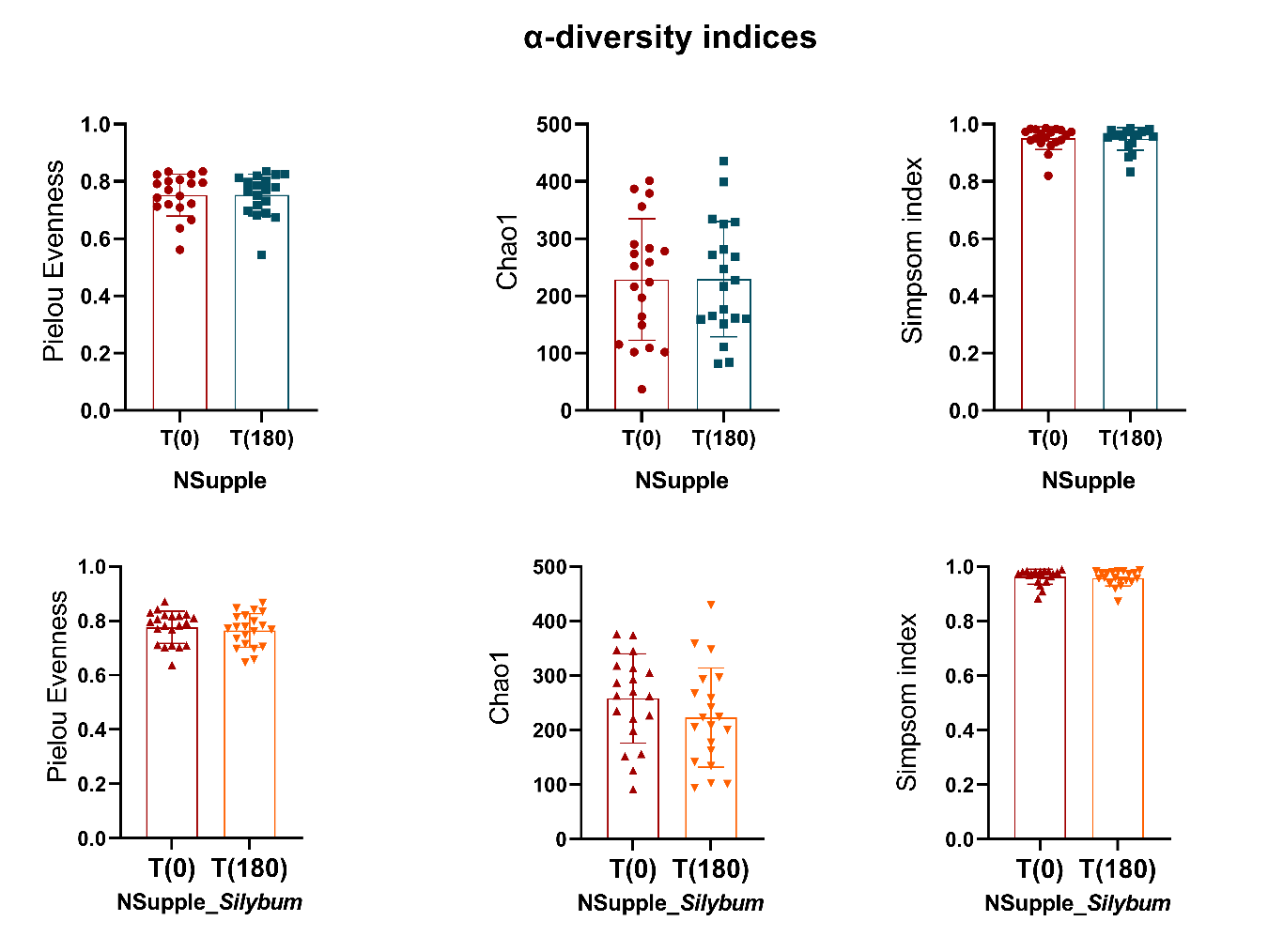


**Figure 1S.** Alpha (α)-diversity indices of the gut microbiota in overweight volunteers after 180 days of supplementation. Shown are the α-diversity indices for species richness (Chao1), evenness (Pielou evenness), and diversity (Simpson) in fecal samples from time zero [T(0)] and 180 days [T(180)] after supplementation with NSupple (n=21) and NSupple_Silybum (n=20) supplement in overweight volunteers. Values are expressed as the percent of relative abundance (mean ± standard deviation).
